# Multiple mechanisms and applications of tertiary lymphoid structures and immune checkpoint blockade

**DOI:** 10.1186/s13046-025-03318-6

**Published:** 2025-03-05

**Authors:** Zelin Li, Shuhan Liu, Deyu Liu, Kangping Yang, Jing Xiong, Ziling Fang

**Affiliations:** 1 The 1st Affiliated Hospital, Jiangxi Medical College, Nanchang University, Nanchang, China; 2 Department of Clinical Medicine, Queen Mary School of Nanchang University, Jiangxi Medical College, Nanchang University, Nanchang, China; 3 The 2st Affiliated Hospital, Jiangxi Medical College, Nanchang University, Nanchang, China; 4 Department of General Practice, The 1st Affiliated Hospital, Jiangxi Medical College, Nanchang University, Nanchang, China; 5 Department of Oncology, The 1st Affiliated Hospital, Jiangxi Medical College, Nanchang University, Nanchang, China

**Keywords:** Immune checkpoint blockade, Immune escape, Tertiary lymphoid structures, Immune-related adverse events, Immunotherapy, Cancer

## Abstract

**Background:**

Immune checkpoint blockade (ICB) inhibits tumor immune escape and has significantly advanced tumor therapy. However, ICB benefits only a minority of patients treated and may lead to many immune-related adverse events. Therefore, identifying factors that can predict treatment outcomes, enhance synergy with ICB, and mitigate immune-related adverse events is urgently needed.

**Main text:**

Tertiary lymphoid structures (TLS) are ectopic lymphoid tissues that arise from the tumor periphery. They have been found to be associated with better prognosis and improved clinical outcomes after ICB therapy. TLS may help address the problems associated with ICB. The multiple mechanisms of action between TLS and ICB remain unknown. This paper described potential mechanisms of interaction between the two and explored their potential applications.

## Introduction

Immunotherapy is emerging as a major cancer treatment strategy. Immune checkpoint blockade (ICB) therapy has demonstrated promising outcomes in treating several malignancies, including melanoma [1], non-small cell lung cancer (NSCLC) [2], thymoma [3], head and neck cancer [4], and gastrointestinal tract cancer [5], making it a mainstay of tumor immunotherapy. Even chemotherapy-resistant cancers and metastatic tumors have demonstrated significant therapeutic responses. Responses to immune checkpoint inhibitors (ICIs) are long-lasting and may persist for years without continued treatment. Since the approval of cytotoxic T lymphocyte-associated protein 4 (CTLA-4) inhibitors for the treatment of metastatic melanoma in the United States in 2011, *PD-1/L1* inhibitors have been used to treat over 20 other malignancies [6]. However, despite significant improvements in patients during the early stages of ICI therapy, disease recurrence seems inevitable. Few patients experience a durable response, and most eventually develop drug resistance [7]. Additionally, non-specific immune activation induced by ICB may lead to immune-related adverse events (irAEs) [8]. Many patients treated with ICB exhibit severe effects on the skin and its appendages [9], digestive system [10], nervous system [11], musculoskeletal system [12], and even lesions in the pancreatic islets [13]. Drug resistance and irAEs limit the broader application of ICB therapies, emphasizing the need for strategies to address these challenges.

Tertiary lymphoid structures (TLS) are ectopic lymphoid tissues present in inflammatory, infected, or neoplastic tissue. The rate of intratumoral TLS production has been associated with a positive prognosis in many tumors, including breast cancer [14], ovarian cancer [15], NSCLC [16, 17], pancreas cancer [18], gastric [19], melanoma [20], and oral cancer [21]. Intratumoral TLS can exert a direct antitumor effect by enhancing immune cell maturation and antibody production. Additionally, TLS may play a role in cancer therapy alongside ICB, as ICB therapy may induce the production of TLS in tumors. A high intratumoral prevalence of TLS is associated with a low incidence of irAEs. These observations have prompted researchers to explore TLS in predicting the degree of ICB resistance and prognosis and investigate ICB-TLS synergistic therapy.

TLS may improve ICB effectiveness to benefit more patients with cancer and those with other immune diseases. However, many potential links between TLS and ICB have yet to be fully explored. Therefore, this paper presented a detailed and clear description of the various mechanisms linking TLS and ICB and explored potential future therapeutic options.

## Mechanisms of ICB resistance

Resistance to ICBs is broadly classified into two categories: (1) primary resistance, which is the total absence of initial tumor response to ICBs [22]. (2) acquired resistance, which is when a patient initially responds to ICB treatment for a period of time but eventually develops clinical and/or radiologic disease progression. The main cause of primary resistance is somatic DNA aberrations, which affect the number and quality of immune effector cells in the tumor microenvironment (TME) and the expression level of tumor-associated antigens. For example, the lack of *PD-L1* expression or T cell activation in tumor cells prevents their migration and infiltration into the TME [22]. These changes prevent an individual from responding to the first immunotherapy, resulting in rapid disease progression. Patients with acquired resistance initially respond to ICBs but eventually develop resistance. After ICB treatment, some responders become non-responders. Clinical data from a melanoma trial using *anti-PD-1* inhibitors revealed that most of the patients developed acquired resistance after achieving complete or partial remission [23].

In acquired resistance, the immune system first succeeds in recognizing and combating tumor cells; however, over time, tumor cells adapt to the immune attack and develop resistance. Tumor cells and TME undergo changes after interacting with the immune system, which confers them with new features and protects them from immune cells. This process can be explained by changes in tumor function, inhibitory checkpoint protein expression, and TME composition. Additionally, tumor cells may evade the immune system, resulting in the selection of inherently drug-resistant cells, which results in drug resistance throughout the tumor [24].

The molecular basis of ICB resistance is complex. For primary resistance, somatic cell DNA aberrations affect the expression levels of tumor-associated antigens in the TME and the number and quality of immune cells. The mechanisms of acquired resistance are categorized into three types: (1) Tumor-associated gene mutations: For example, *JAK1/2* loss-of-function mutation in tumor cells can disrupt the interferon-gamma (IFN-γ) signaling pathway terminal, reducing immune cell activation and infiltration [23]. (2) Increased expression of ICIs: For example, increased expression of LAG-3 (lymphocyte activation gene 3 (LAG-3, CD223), T cell immunoglobulin 3 (TIM-3, CD366), and T cell immunoglobulin and ITIM structural domain proteins (TIGIT) in tumor cells or immune cells causes enhanced immunosuppressive signaling, which can weaken the efficacy of ICBs. (3) Increased immunosuppressive components in the TME: Myeloid-derived suppressor cells decrease the ratio of CD8^+^ T cells to Tregs, inhibit the cell cycle of T cells, and contribute to tumor aggregation. The aggregation and activation of Tregs in the TME suppress effective immune responses. Additionally, ecological dysregulation of the gut microbiome and the combination of radiotherapy with immunotherapy can contribute to acquired resistance to ICB [23].

### Deficiencies in antigen presentation mechanisms

**Fig. 1:**
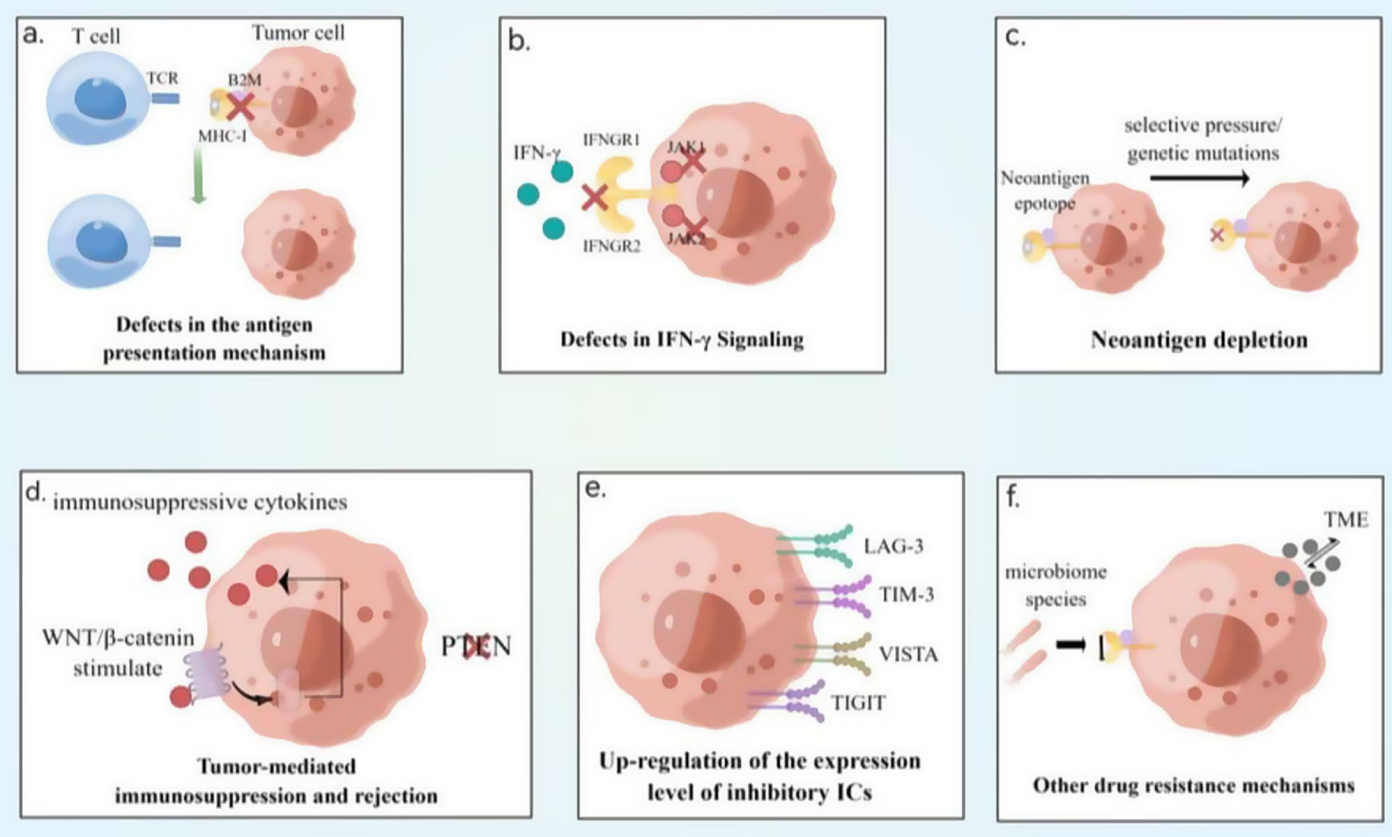
**(a)** Defects in antigen presentation mechanisms, such as inhibition of *MHC-I* binding to TCR on the surface of tumor cells. **(b)** IFN-γ signaling pathway plays an important role in immune response, whereas defects in *IFNGR1*, *IFNGR2*, and *JAK-related genes* lead to abnormalities in the IFN-γ signaling pathway. **(c)** A decrease in neoantigenic epitopes due to selective pressure/gene mutations leads to neoantigen depletion. **(d)** Activation of the WNT/β-catenin pathway leads to the formation of large amounts of immunosuppressive cytokines, which promotes tumor progression and stimulates tumor-mediated immunosuppression and rejection, just like PTEN deletion. **(e)** Upregulation of the expression levels of immune checkpoints, such as LAG-3, TIM-3, VISTA, and TIGIT, further suppresses immune responses. **(f)** Species changes in the microbiome and changes in the TME affect tumor development as other mechanisms of drug resistance. In summary, tumor cells evade the surveillance and attack of the immune system via multiple pathways, resulting in the development of immunosuppression and drug resistance

Major histocompatibility complex (MHC) recognition of antigens on antigen-presenting cells (APCs) is necessary for the activation of T cell-mediated immunity. MHC-I delivery of tumor antigens is mediated by the co-expression of several genes, including β2-microglobulin (*B2M*), a major component of MHC-I that presents antigens to CD8^+^ T lymphocytes **(**Fig. 1a**)**. Tumor-specific CD8^+^ T lymphocytes are unable to detect tumor cells because of mutations in the *B2M* gene. In many acquired drug resistance studies, some individuals who received *PD-1* blockade exhibited truncated changes in *B2M* [7]. Additionally, we speculate that many double allelic changes are associated with loss of expression of the *B2M* and MHC-I-like molecules according to the relevant immunohistochemistry studies [25]. However, it is unclear whether changes in a single allele of the *B2M* gene contribute to ICB resistance. The expression of MHC-I proteins and resistance to ICBs may be regulated by other unidentified genomic or non-genomic mechanisms [24].

### IFN-γ signaling

IFN-γ plays a significant role in antitumor immunomodulation. It can increase the sensitivity of cancer cells to apoptosis, inhibit the proliferation of endothelial cells in the TME, prevent tumor angiogenesis, and increase MHC expression on APCs [26]. IFN-γ is used clinically in the treatment of certain cancers and plays a role in antitumor immunomodulation by increasing the sensitivity of cancer cells to induced apoptosis and inhibiting the proliferation and survival of endothelial cells in the TME [27]. Additionally, it prevents tumor angiogenesis and enhances MHC expression on APCs. IFN-γ released from effector T cells initiates a signaling cascade in tumor cells via the *JAK-STAT* pathway, regulates *PD-L1* and MHC-I expression, and induces tumor cell death in a variety of other ways [28]. The binding of IFN-γ to the heterodimeric *IFNGR1/IFNGR2*, which activates the receptor-associated kinases *JAK1* and *JAK2*, is a key step in this pathway **(**Fig. 1b**)**. Clinical studies have revealed that individuals with *JAK1* or *JAK2* inactivation have loss-of-function mutations in the *JAK1* or *JAK2* genes. These mutations are located upstream of the structural domain of the kinase and may result in truncation or nonsense-mediated decay of the *JAK* protein. A study collected tumor cells from patients who had acquired the *JAK2* mutation and created cell lines that demonstrated that the mutation resulted in complete deletion of the *JAK* protein and decreased sensitivity of the tumor cells to IFN-γ, which subsequently resulted in the downregulation of MHC-I and *PD-L1* expression in the tumor cells [28]. *In a clinical study of paired baseline and recurrent lesion biopsies from four patients with PD-1-treated metastatic melanoma*,* the investigators found that loss-of-function mutations in JAK1 and JAK2 resulted in no response to interferon γ*,* while truncating mutations in B2M resulted in the loss of surface expression of the MHC class I molecule*,* revealing the emergence of acquired drug resistance to anti-PD-1 treatment in melanoma* [29]. These findings suggest that ICB treatment-induced imbalance in the IFN signaling pathway partially contributes to the emergence of acquired drug resistance. However, the link between genomic alterations in the IFN-γ pathway other than *JAK1* and *JAK2* and drug resistance in ICBs is unclear [7]. *In addition*,* studies indicate thatcancer patients exhibit exhaustion of CD8*^*+*^*T cells due to the development of ICB resistance* [23] *upon treatment with PD-1 inhibitors*,* which consequently leads to T cell exhaustion. As a key cell in the immune system*,* CD8*^*+*^*T cells produce cytokines that can influence the structure and function of TLS. Therefore*,* we speculate that ICB resistance may also impact the functionality of TLS*,* diminishing the antitumor immune response.*

### Neoantigen depletion

Neoantigens are antigens that are newly produced in tumor cells because of many tumor-specific alterations (DNA mutations, dysregulated RNA splicing, and impaired post-translational modifications). The quantity of these antigens correlates with the level of inflammation in the TME. High levels of CD8^+^ T cell infiltration are usually centered around neoantigens [30]. These neoantigen-specific T cells may be key regulators of the immune response to ICBs. Thus, immune escape from tumors may be caused by the loss of genes encoding tumor-specific neoantigens (through clonal selection, epigenetic suppression, or copy number loss) **(**Fig. 1c**)**. In a clinical report of four patients with NSCLC resistant to ICB, no loss-of-function mutations in HLA genes such as *JAK1*, *JAK2*, or *B2M* were identified. However, a comparison of pre- and post-treatment data revealed that many of the altered genes might initially be capable of producing neoantigens. The two processes of neoantigen loss in acquired drug-resistant cancers that have been described to date are as follows: (1) tumor cells with neoantigens are cleared by the immune system, while cells lacking neoantigens are selected for and rapidly proliferate; (2) gene mutations in tumor cells directly result in the loss of neoantigens [7, 31].

### Tumor-mediated immunosuppression and rejection

In preclinical models, the absence of the tumor suppressor *PTEN*, which is essential for controlling phosphatidylinositol 3-kinase activity and thus inhibiting T cell-mediated infiltration, was observed by increasing the expression of immunosuppressive cytokines and decreasing the T cell effector IFN-γ. Besides, the absence of *PTEN* was observed in cases of acquired resistance. Similar to *PTEN* loss, stimulation of the WNT/β-linker pathway promoted tumor escape by preventing TIL and CD103^+^ dendritic cell recruitment into the TME **(**Fig. 1d**)** [32]. *PTEN* gene deletion and activation of the β-catenin signaling pathway are two oncogenic abnormalities associated with insufficient T cell infiltration at tumor sites. These alterations promote acquired resistance in patients with metastatic melanoma after combination ICB therapy [33, 34].

### Upregulation of expression levels of ICIs

According to several findings, the expression of other T cell checkpoints, for example, LAG-3, TIM-3, TIGIT, and VISTA, was elevated during acquired resistance **(**Fig. 1e**)**. This demonstrates a different approach to resistance development [23].

Only T cells produce LAG-3, which binds MHC-II more strongly than other cell types and inhibits MHC-II-mediated antigen presentation, antigen-specific CD4^+^ efferent T cell responses, and CD4^+^ T cytokine production. Inhibition of monocyte development into macrophages and DCs and promotion of Treg differentiation prevent immunostimulation [35].

TIM-3 is a transmembrane protein expressed in CD8^+^ cytotoxic T cells and CD4^+^ Th1 cells, Treg cells, NK cells, DC cells, macrophages, and monocytes. In melanoma patients, the anti-TIM-3 antibody restored the T cell function of TIM-3^+^CD4^+^ and CD8^+^ cells, and these cells contribute to the TME [36], which suggests a certain connection between TIM-3 and cancer progression.

VISTA is an Ig inhibitor of the V structural domain of T cell activation. It is also known as *PD-1 homolog (PD 1 H)* and belongs to the B7 family of proteins. It encodes a type I membrane protein expressed in hematopoietic cells. Additionally, it inhibits early T cell receptor (TCR) activation and blocks the cell cycle, negatively regulating CD4^+^ T cell responses. Furthermore, it prevents the production of IL-2 and IFN-γ by CD8^+^ T cells. The latter is critical for T cell survival and growth. VISTA expression is increased in patients with melanoma treated with *anti-PD-1* therapy, suggesting a potential link between acquired drug resistance and cancer [37].

TIGIT is a co-inhibitory receptor expressed on lymphocytes belonging to the poliovirus receptor vPVR/adhesion protein family. It has a high affinity for CD155-CD112 and competes with CD226 [36]. Its binding disrupts the activation of co-stimulatory signaling pathways. TIGIT expression is elevated in CD8^+^ T cells of several malignant tumors, and a high TIGIT/DNAM1 ratio in Treg correlates with poor prognosis after blockade of the *PD-1* and/or CTLA-4 pathways. In particular, TIGIT is involved in the control of antitumor immunity mediated by tumor-infiltrating microbes. In the TME, TIGIT and *PD-1* synergistically inhibit the activity of CD226, thereby limiting the anti-tumor immune response of T cells [38].

### Other resistance mechanisms

There is growing evidence that changes in the TME and microbiome species and abundance are associated with immune responses to cancer **(**Fig. 1f**)**. By improving effector T cell function in the TME and facilitating antigen presentation, a fraction of the gut flora can contribute to anti-cancer immune responses. Other components of the gut microbiota negatively affect intratumoral lymphoid infiltration and antigen presentation [39]. Clinical evidence suggests that in a cohort of patients with advanced renal, pulmonary, and uroepithelial malignancies treated with *anti-PD-1* therapy, those who did not receive antibiotics survived longer than those who did. Additionally, patients deficient in bacteroides fragilis demonstrated a poorer immune response to anti-CTLA-4 therapy [40].

Cancer cells can influence the TME by secreting cytokines, chemokines, and other factors. T cell-mediated antitumor immune responses are critical for immunotherapeutic efficacy, and their dysfunction is at the core of ICB resistance. The TME is known for its hypoxic and low-glucose environments; however, glucose deprivation is detrimental to T cell activation and limits antitumor immunity. Meanwhile, certain TME components can promote extracellular matrix remodeling, facilitate tumor metastasis and T cell inactivation, and induce resistance to ICBs [39].

In most cases, it is difficult to identify specific mechanisms of acquired resistance. While some clinical papers provided overlapping explanations or implicitly inferred mechanisms of acquired resistance from the data, others did not explore specific processes. Overall, both primary and acquired resistance requires more research [7].

Overall, most patients with tumors develop resistance to ICBs, and tumor cell escape is a key factor in the mechanism of ICB resistance. Considering the existing research progress, we propose that, in addition to focusing on the known immune cells and tumor cells, attention should be given to the role of other cells in the TME, such as fibroblasts and endothelial cells, in ICB resistance. Understanding their relationship with the composition and function of the TLS may provide clues for further investigating the mechanism of ICB resistance and developing new therapeutic strategies.

## TLS

### Cellular composition of TLS

TLS are commonly found in inflammatory, infected, or neoplastic tissues. Their structural composition is very similar to that of lymph nodes (LN), and they are associated with the generation of an adaptive immune response [41]. The organization and integrity of stromal cells in TLS include T cell areas containing mature DCs, follicular dendritic cells (FDCs), germinal centers (GCs) containing proliferating B cells, high endothelial venules (HEVs), and fibroblasts [42]. Well-developed TLS contain B cell follicles in which the T cell region surrounds the B cell germinal centers and actively replicates around them. Simultaneously, HEVs and DC lysosome-associated membrane protein (LAMP^+^) cells are dispersed in the TLS, forming a complex network of non-hematopoietic stromal cells [43].

In humans, B cells are the major cellular component of TLS. B cells are present in most types of cancer and are usually associated with favorable clinical outcomes [44]. Notably, structural analysis revealed that CD20^+^ B cells were located in the TLS of responders’ tumors and co-localized with CD4^+^, CD8^+^, and FOXP3^+^ T cells, CD21^+^ FDCs, and MECA79^+^ HEVs [45]. The spatial analyses that have been performed to date have identified more activation markers for T cells within the TLS than for T cells outside the TLS, which may reflect a potential functional role for B cells and the TLS in promoting T cell responses [46]. Furthermore, CXCL13^+^ CD103^+^ CD8^+^ tissue-resident memory T cells are present in TLS, which may play an important role in antitumor immunity [47].

### TLS formation

**Fig. 2:**
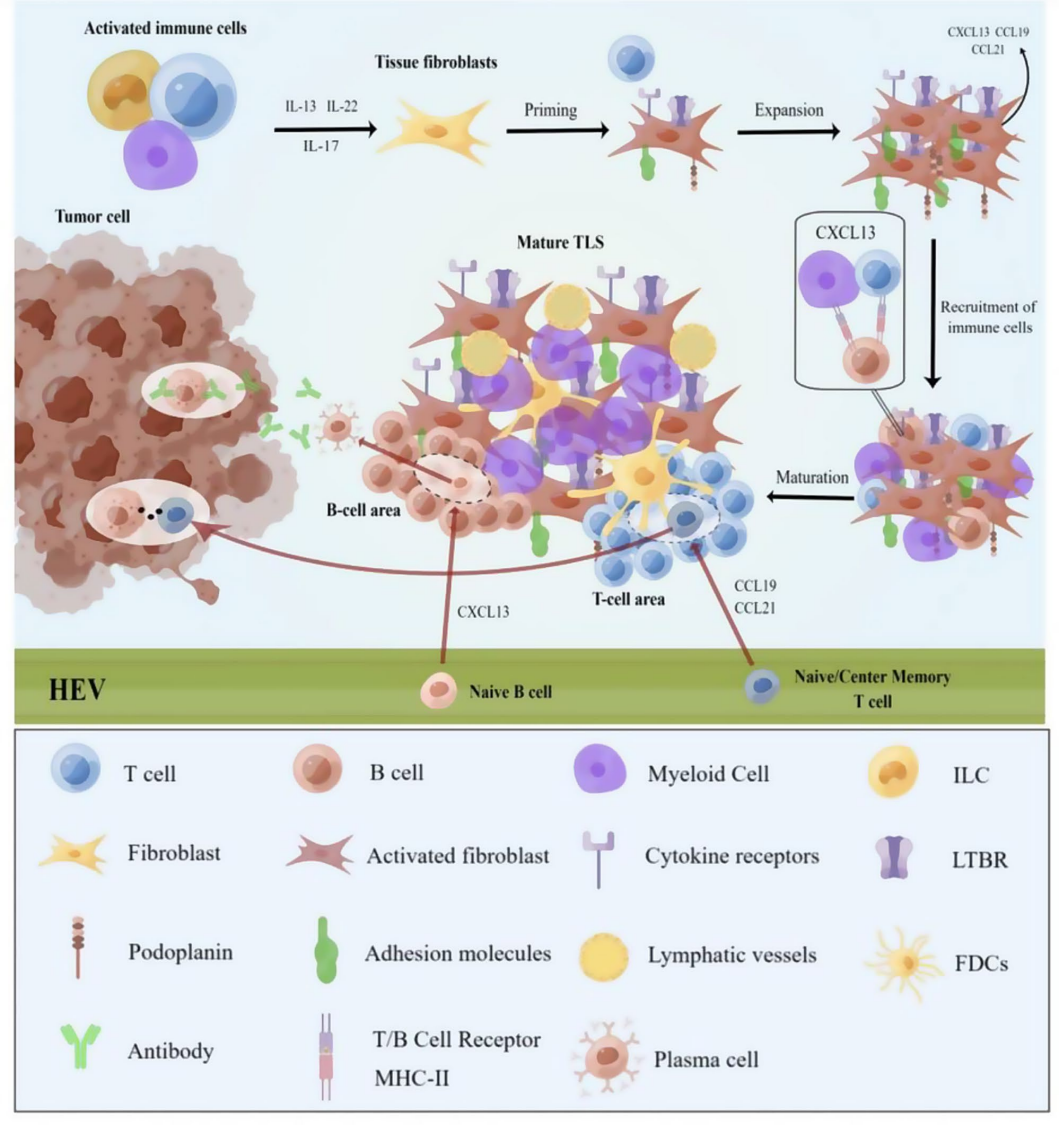
Activated immune cells activate tissue fibroblasts through a variety of cytokines, including IL-13, IL-22, and IL-17. The initiated fibroblast molecules contain adhesion molecules, cytokine receptors, LTBR, and podoplanin proteins, which continue to expand and undergo immune cell recruitment to promote TLS maturation. The mature TLS contains a B and a T cell region, with different regions having different functions; naïve B cells differentiate to generate plasma cells, which produce antitumor antibodies in tumor cells, while T cells are responsible for immune recognition and attack

TLS are associated with persistent bacterial and viral infections and are commonly found in chronic inflammatory environments. During acute infection, TLS in infected tissues can generate an adaptive immune response that promotes pathogen clearance. Conversely, during chronic infection, the local inflammatory cytokine and chemokine environment favors the initial production of TLS [48]. Additionally, TLS have been reported in the synovial lymphoid tissue of patients with rheumatoid arthritis (RA). These TLS not only amplify the local immune response but also play a crucial role in the pathogenesis of RA by supporting the generation of autoantibodies, such as rheumatoid factor and anti-citrullinated protein antibodies, which are closely associated with disease severity and progression [49]. The formation of TLS can be divided into the following stages: (1) Fibroblast activation: fibroblast activation is a critical stage in TLS development. Tissue fibroblasts provide the ecological niche for chemokines that attract and organize hematopoietic cells during inflammation. Several pathways can stimulate tissue fibroblasts. For example, both IL-13 and IL-17 can activate tissue fibroblasts [50, 51]. Afterward, myeloid cells, CD4^+^ T cells, and innate lymphoid cells (ILCs) can activate local tissue fibroblasts [52]. (2) Immune cell recruitment: stromal and hematopoietic cells can produce chemokines that attract lymphocytes to tissues [53]. (3) TLS maturation: developing FDCs within B cell follicles is a feature of TLS maturation [54]. Prolonged inflammatory conditions further mature TLS structures and can attract naïve cells **(**Fig. 2**)**. Hepatitis E virus must be formed during TLS development for more lymphocytes in the bloodstream to enter the TLS. HEV development appears to be dependent on lymphotoxin-α1β2 (LTα1β2) signaling and, to a lesser extent, on lymphotoxin beta receptor(LTBR) [55].

TLS production is a multistep process in which immune cells provide proinflammatory signals to drive TLS formation, activate fibroblasts to construct and maintain lymphoid ecological niches, and immune cells are attracted to allow chemokines to function as organizers. The first aggregation of immune cells can be understood to be initiated by a re-enforcement of fibroblast activity and additional maturation of the TLS through local antigen presentation [56]. Furthermore, TLS may be the site of an amplified autoimmune response in which inappropriate (tissue damage and/or sustained) T and B cell activation may occur, leading to autoimmune or other harmful diseases. In the laboratory, researchers have demonstrated mechanisms to induce TLS using cytokines or chemokines that control the development of secondary lymphoid organs (SLOs) [57]. Notably, the formation of TLS occurs not only in the chronic inflammatory environment but may also be induced in the TME. Certain therapeutic interventions, including ICI therapy, may promote TLS formation in tumors [58].

### TLS maturation

TLS develops through different stages of maturation, from an initial aggregation of only a few T cells to compartmentalization of B cells and T cells with FDC networks [59]. Immature TLS contain many T and B cells but no B cell follicles and FDCs, indicating that immature or memory B cells do not mature further. These immature TLS are referred to as early TLS; however, not all these lymphoid aggregates develop to a more mature stage [60]. Some will remain immature, possibly because of a lack of sustained antigenic stimulation and/or associated chemokines and cytokines. In more mature TLS, CD21^+^ FDCs may be involved in the presentation of antigens to B cells, resulting in further maturation of B cells and the formation of primary follicles lacking GCs. Conversely, fully mature TLS contain GCs with CD21^+^ CD23^+^ FDCs, which may provide antigens for screening B cells with high affinity for B cell receptors (BCRs) and T follicular helper cells (Tfh) [56]. New studies have revealed that mature TLS (especially those containing germinal centers) are associated with a better prognosis and immunotherapeutic response. In NSCLC, TLS maturation may be affected by tumor-draining (TD)LN, and TDLN metastasis may reduce germinal center formation in TLS, which is associated with memory B cell differentiation and the IFN-γ signaling pathway [61].

Mature TLS identification is easily achieved in standard pathology laboratories using a combination of CD20, CD3, and CD23 markers. Additionally, multiplex bright-field immunohistochemistry and/or multiplex immunofluorescence analyses are able to detect the hallmarks of mature TLS, namely, the FDC network in GCs [62]. We believe that in the future, advances in researchers’ understanding of the regulation of mature TLS development and function, as well as factors controlling B and T cell differentiation in TLS, including FOXP3, CXCL13, and BCL6, will further improve the accuracy of TLS identification and refine TLS classification.

TLS are commonly found in inflammatory tissues, and their components include DCs, FDCs, GCs, HEVs, and fibroblasts. Different cell types interact with each other via different mechanisms, and these mechanisms of action affect the role of TLS in tumor immune response. *B cells are the major cellular component of TLS*,* mature TLS are often characterized by a high density of B cells*,* which is usually associated with favorable clinical outcomes* [44]. TLS formation is a multistep process that includes three stages: fibroblast activation, immune cell recruitment, and TLS maturation. *Mature TLS are associated with a better prognosis and immunotherapeutic response* [44]. Therefore, finding drugs or biologics that can promote TLS formation and maturation may enhance the effect of TLS on the immune response to tumors.

## TLS in Oncology

TME shares many similarities with chronic inflammation; however, it also exhibits profound immunosuppressive features, including infiltration of myeloid-derived suppressor cells, Treg cells, and M2 macrophages, which results in tumor metastasis and reduced chemo/radiotherapy efficacy in most cases [63]. Therefore, some researchers suggested that TLS are induced and maintained by tumor-associated chronic inflammation, serving as passive witnesses to antitumor immunity or sites of memory lymphocyte reactivation. However, the TME component that induces TLS development has not been fully defined.

When studying TLS, we must examine the cellular composition of the TME and its spatial organization. Some tumors exhibit no immune cell infiltration in the TME, or immune cells infiltrate the tumor but fail to form an organized network, while other tumors have a TME with immune cells that form and function as TLS. Currently, researchers use mouse models to investigate the role of TLS in antitumor immune responses [64]. However, most of these models are based on cultured cell lines, and the resulting tumors rarely form TLS. *Nevertheless*,* TLS formation has been observed in several spontaneous tumor models* [64]. One possible explanation is that the rapid growth of transplanted tumors inhibits TLS formation; however, this hypothesis requires further confirmation through additional studies.

In mature TLS, both plasma and effector T cells are involved in antitumor immunity. *Analysis of RNA sequencing (RNA-seq) data of B cell receptor sequences using a modified TRUST algorithm revealed increased BCR diversity and clonal counts of heavy- and light-chain immunoglobulins in responders compared with non-responders* [46], *implying that antitumor antibodies can be produced within tumors*,* possibly by TLS-producing plasma cells*,* suggesting that B cells play an active role in antitumor immunity.*

CD8^+^ T cells are heavily infiltrated in tumors with a high tumor mutational burden (TMB), including NSCLC, melanoma, and microsatellite-unstable colorectal cancer (CRC). TMBs vary across cancers and are often used as a proxy for the frequency of MHC-presented epitopes on tumor cell surfaces. *These epitopes have been associated with CD8*^*+*^*T cell infiltration*,* particularly memory CD8*^*+*^*T cells with cytotoxic activity. These cells are often associated with longer progression-free survival (PFS) and overall survival (OS)* [44], *as they play an immunosurveillance role by recognizing tumor-specific mutant antigens.* Although the presence of CD8^+^ T cells is crucial, they alone are insufficient to induce a sustained antitumor immune response. CD8^+^ T cell-dependent mechanisms are required to recruit LTα1β2-secreting B cells, which act as lymphoid tissue-inducing cells alongside CD8^+^ T cells to orchestrate TLS formation and development [44, 65]. In tumor-adjacent TLS, some B cells are in close proximity to CD8^+^ T cells, potentially co-regulating the overall antitumor immune response. However, the relationship between tumor-associated TLS and humoral immune responses is unclear and requires further investigation [42].

TLS play distinct, important roles in different TMEs, and these roles may vary depending on the tumor type and stage. The following is a comparative analysis of the specific roles of TLS in different tumors: (1) Hepatocellular carcinoma (HCC): HCC is an inflammation-driven cancer characterized by many hepatic sclerosis nodules with TLS in non-tumor tissues. TLS have a dual role in HCC [64]. The expression of a 12-gene TLS signature and the detection of TLS in tumor-adjacent liver tissue have been linked to an increased risk of late recurrence. Additionally, the risk of tumor recurrence is linked to TLS location. TLS in the tumor core are associated with a low risk of early recurrence, reflecting persistent and effective antitumor immunity. Conversely, TLS located in the tumor periphery are associated with a good prognosis, whereas TLS farther from the tumor are a marker of poor prognosis [44] and negatively correlate with TLS maturity. (2) Lung cancer: TLS can control tumor invasion and metastasis, and their presence positively correlates with the survival and the disease-free survival of patients with lung cancer. In lung cancer, TLS are directly associated with somatic hypermutation, class switching, and the production of a wider range of tumor-specific antibodies by plasma cells within the tumor [64, 66]. TLS presence in lung cancer is associated with favorable prognostic outcomes independent of pathologic staging [63]. (3) *CRC: Studies have shown that the density of TLS in CRC is positively correlated with better survival rates and negatively correlated with tumor staging* [67, 68]. Despite a correlation between TLS density and infiltrating T cells, the favorable prognostic value of both is independent of one another in stage II CRC [64]. (4) Pancreatic cancer: In a neuroendocrine pancreatic cancer mouse model, the combination of antiangiogenic and *anti-PDL1* therapies increased HEV formation and subsequent TLS formation [64]. CD8^+^ and CD4^+^ T cells in TLS demonstrated favorable prognoses for most tumors. *PD-L1* blockade, combined with anti-angiogenic therapy, resulted in the conversion of tumor vasculature into HEVs and subsequent TLS formation. The increased CD8^+^ T cell stimulation and tumor destruction was associated with TLS-induced persistent antitumor immune responses throughout the body. Tumor-infiltrating B cells (TIL-B) are an important component of TLS, and TIL-B cells in TLS have been linked to an improved survival prognosis in patients [69]. Therefore, TLS has a generally favorable effect on survival in patients with pancreatic cancer. (5) Breast cancer (BC): The presence of TLS is typically associated with an increased immune infiltration, particularly in triple-negative breast cancer. There is a sustained B cell response and TLS development in BC, as revealed by scRNA-seq analysis of B cells. The genetic characterization of 12 chemokines in TLS, particularly CCL19, CCL21, and CXCL13, has been linked to increased survival in patients with BC, CRC, and other cancers [64]. Furthermore, the role of TLS in metastatic and primary tumors varies across cancer types. For example, CRC lung metastases exhibit well-developed TLS, whereas RCC lung metastases *do not* [70]. In a study of patients with melanoma, TLS was absent in primary tumors but present in metastases.

Overall, the role of TLS in different tumors is complex and may vary depending on tumor type and stage. TLS is associated with patient prognosis; however, in some cases, such as high-grade tumors or TLS in the presence of tumor cells, its prognostic value may be reduced. Therefore, the specific role of TLS in the TME requires further investigation for better understanding.

*Although TLS have been detected in many cancers*,* there is currently no standardized methodology for measuring and assessing TLS.* Different studies may use different techniques to quantify TLS, which may result in reduced comparability of results. Previous studies evaluated the density of DC-LAMP^+^ cells to predict TLS prognosis. Recent studies have revealed that CD20^+^ aggregates have similar prognostic significance to CD8^+^ aggregates. However, using only a single parameter to quantify cells in TLS may produce quite different results from those obtained from Hematoxylin and Eosin (H&E) calculations of total aggregate densities [70]. Owing to the lack of consensus on what constitutes a “high” or “low” density, it is difficult for us to quantify the number of cells in TLS [70]. Although H&E staining allows for the direct quantification of TLS as dense aggregates of lymphocytes with distinct contours compared to unorganized inflammation [71], objective and reliable comparisons of TLS development across different studies are hampered by the wide variation in the definition of TLS in H&E staining. In addition, the detection of various TLS-associated cell types, such as B cells (CD20), DC-LAMP^+^ cells, HEVs, or T cells (CD3, CD8) [71], *by immunohistochemistry to quantify TLS also presents difficulties. Understanding the implications of TLS density remains a challenge.* Consequently, the lack of a consensus methodology makes it difficult to standardize the use of TLS density, limiting its widespread use as a prognostic marker for tumors. The development of standardized methods for measuring the number and composition of TLS could help further reveal the predictive and prognostic assessment value of TLS in different diseases.

Based on the current understanding of the role of TLS in oncology, some potential innovative directions can be explored. By monitoring the dynamic changes of TLS in tumor immunotherapy, we can more accurately assess the therapeutic effects and predict patient prognosis. Additionally, we can enhance immune cell activity by modulating T cell formation and function and investigate TLS differences in different tumor types and the effects of TLS on tumor treatment to develop personalized tumor treatment plans.

## TLS and ICB

### ICB induces TLS generation

**Fig. 3:**
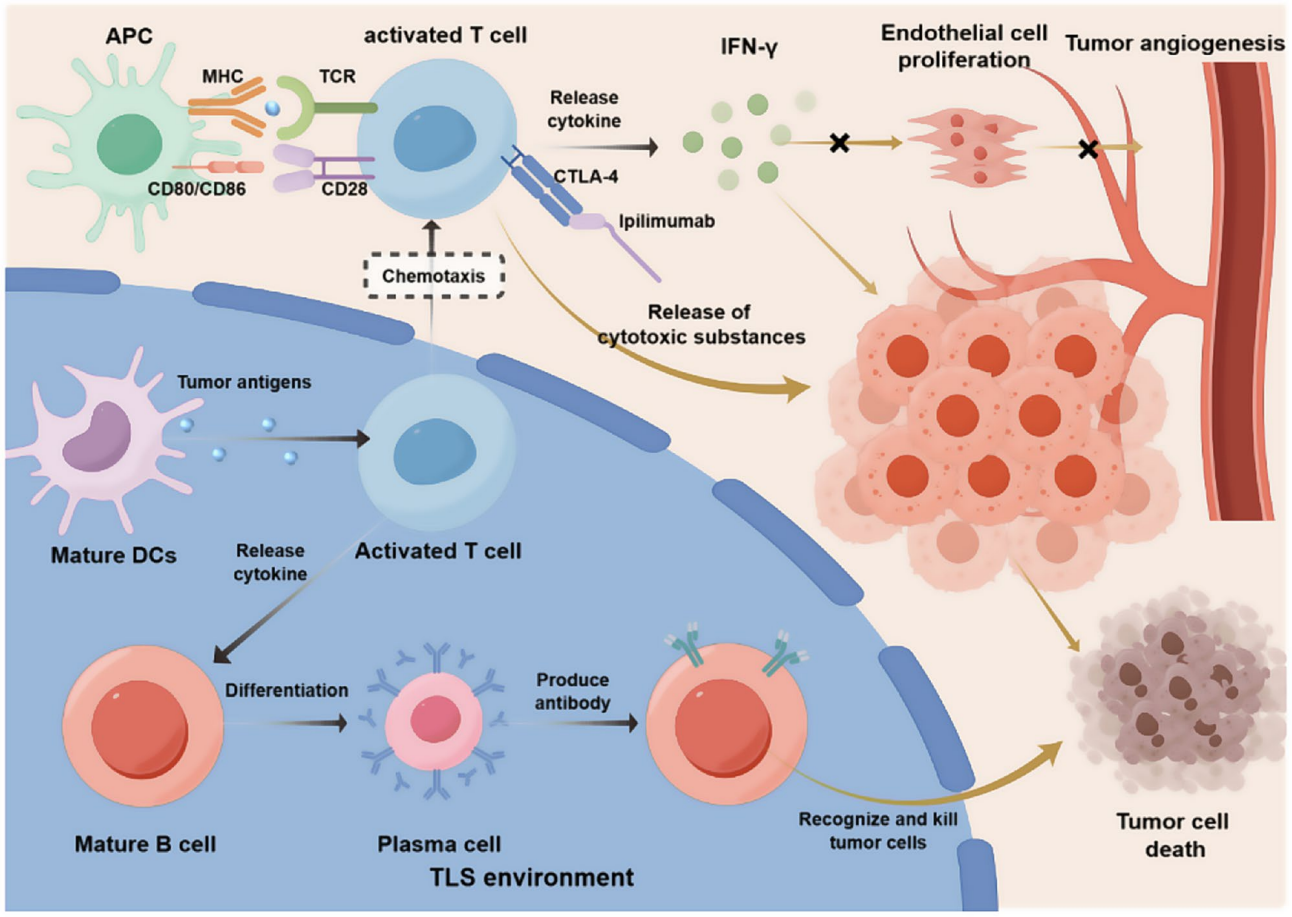
The use of ICB drugs, such as Ipilimumab, to block the immune-checkpoint molecule CTLA-4 can prevent the binding of inhibitory receptors to their ligands and activate T cells. Meanwhile, within the T-cell zone of the TLS, mature dendritic cells can effectively capture, process, and present tumor antigens. By transmitting tumor - antigen signals, they activate T cells and enhance the effect of ICB therapy on T cells, further promoting anti - tumor immune responses. Activated T cells release cytokines (such as IFN-γ), which inhibit the proliferation and angiogenesis of endothelial cells in the tumor microenvironment, increase the susceptibility of tumor cells to apoptosis, and limit the growth and spread of tumor cells. Activated T cells guide B cells to differentiate into plasma cells through the release of cytokines and direct contact, producing anti-tumor antibodies that induce tumor-cell death. It can be seen that the activation and proliferation of immune cells in the TLS provide more active immune-cell targets for ICB. ICB further enhances the functions of immune cells in the TLS by blocking immune-checkpoint molecules, such as PD-1/PD-L1 and CTLA-4, enabling immune cells to more effectively identify and attack tumor cells. This synergy can enhance the overall immune response and improve treatment outcomes. For example, in the treatment of various cancers, combination therapy has significantly extended patients’ median survival time, progression-free survival time, etc

CCL19 and CCL21 recruit naïve/central memory T cells to tumor tissue [72], allowing them to migrate from the HEVs into the TLS. Mature DCs in the T cell compartment of the TLS can initiate/activate an immune response that can spread to the tumor site and destroy the tumor cells. CXCL13 attracts naïve B cells to the TLS-B cell zone, similar to naïve/central memory T cells, where they interact with Tfh and FDC to continue maturation [73, 74]. Mature B cells can differentiate into memory B cells, circulating plasma cells (PCs), or antibody-producing intratumoral PCs. These mature B cells produce antibodies that bind to tumor antigens and induce tumor cell death. In mouse models, anti-PD-1 monotherapy or combination therapy with anti-CTLA-4 significantly increased the size and number of TLS and enhanced their microanatomical organization into distinct T cell and B cell/FDC regions [75].*This observation suggests that ICB has a strong impact on tumor-associated TLS and may enhance antitumor immune responses by increasing the number and size of TLS. As mentioned above*,* ICB can induce the generation of TLS*,* and TLS affects the therapeutic effects of ICB through a series of complex mechanisms*,* clearly illustrated in* Fig. 3.

### TLS predicts ICB treatment outcomes

In autoimmune diseases, the presence of TLS is usually associated with increased poor prognostic outcomes; however, in most cancers, tumor-associated TLS are usually associated with good prognostic outcomes. The presence of follicular B cells and Tfh in TLS is associated with a favorable prognosis in breast cancers and the presence of B cells in TLS is associated with protective immunity in lung cancers [76]. TLS allow T cells to differentiate into effector cells upon contact with mature DCs and B cells, thereby protecting them from the immunosuppressive effects of TME. Accordingly, TLS are the site of induction and maintenance of local and systemic antitumor responses, and they are associated with better patient prognostic outcomes [77]. Current studies on tumors confirm the ability of TLS to function as a prognostic biomarker for cancer and complement established prognostic immune factors [59]. Additionally, analysis of TLS tissue and density may help identify patients at higher risk of recurrence and guide treatment decisions [70].

In a study of NSCLC tumors, B and plasma cell signaling was enhanced in patients treated with the *PD-L1* inhibitor atelizumab. Patients with TLS- and lymphoid aggregates(LAs)-containing tumors demonstrated a significant increase in OS when treated with atelizumab. This suggests that B cell-rich TLS may contribute to a favorable prognosis with ICB [78]. Studies on soft tissue sarcomas (STS) revealed that, after immunophenotyping of tumors based on the TME, patients with class E tumors exhibited high survival rates in phase II clinical trials and a strong response to *PD-1* blockade. Sarcoma Immune Classes E(SIC E) tumors are characterized by the presence of TLS and are enriched with B cells. It is hypothesized that TLS are sites for antitumor immunity, with TLS-rich tumors more prone to CD8^+^ T cell infiltration. CD8^+^ T cells counteract tumors [79], which explains why ICBs induce stronger antitumor immunity in TLS-containing tumors [80]. Neoadjuvant ICB therapy in patients with melanoma revealed that immune responders demonstrated higher TLS densities and frequencies than non-responders, a feature that was particularly evident in early treatment samples [46]. *In clinical practice*,* before patients undergo ICB therapy*,* it is necessary to first assess the expression level of PD-L1 to evaluate the potential responsiveness to ICB treatment. At the same time*,* by examining the maturity of TLS*,* a comprehensive evaluation of the efficacy of combined therapy can be made. A 58-year-old patient diagnosed with hepatocellular carcinoma (HCC)*,* complicated by portal vein tumor thrombosis (PVTT)*,* liver cirrhosis*,* and chronic viral hepatitis*,* underwent treatment. During the course of therapy*,* research revealed that HCC patients who responded well to combined immunotherapy exhibited specific characteristics in their baseline tumor immune microenvironment (TIME). This discovery suggests that TIME can be utilized to assist in clinical decision-making for HCC immunotherapy* [81]. *In this clinical case*,* high-density TLS were used as a diagnostic criterion for administering camrelizumab in combination with sorafenib or regorafenib to HCC patients with PVTT*,* and a good predicted effect was observed*,* suggesting the value of TLS as a predictive indicator of response to immunotherapy. TLS provides an ordered spatial structure for immune cells*,* enabling more efficient interaction between T cells and B cells. This spatial organization may enhance antigen presentation and immune cell activation*,* thereby improving the immune activity of the TIME* [64].

Although numerous studies have demonstrated that high levels of B cells and TLS improve immunotherapy, the NABUCCO trial discovered that immature TLS were enriched in B cell-related genes in non-CR tumor biopsies compared with those of CR tumors. Research data indicates that in urothelial carcinoma, superficial TLS are located in the submucosal layer of the urothelium, specifically referring to the superficial tissues that are highly exposed to urotoxins, microbial pathogens, and inflammatory mediators.What’s more, a significant difference exists when comparing these superficial TLS with those in deep tissues. The superficial TLS display a much more prominent enrichment of T - helper cells and early - stage TLS [82].Comparisons between superficial and deep TLS revealed that superficial TLS exhibited higher levels of CD4^+^ T cells and a higher proportion of early TLS. This suggests that the superficial TLS composition differs from the deep TLS, which may affect the methodology of immunomarker studies and the identification of predictive [82].

However, if TLS are to be used as a reliable cancer prognostic tool clinically, we need to consider their limitations. A recent study on TLS in rectal cancer discovered that although overall infiltration of CD3 ^+^ T cells was associated with improved patient survival, CD3 ^+^ CD83^+^ lymphoid nodes were prevalent in patients, limiting the prognostic value of TLS [70]. Additionally, the combination of TLS defined in different cancers, including some core components, may vary. In general, TLS contain CD3^+^ T cells, CD20^+^ B cells, DC-LAMP^+^ cells, and HEVs. However, whether they include activated GCs, proliferative B cells, or Tfh remains to be explored across different cancers. Other researchers have suggested that, in addition to CD molecular populations and non-immune components, markers of TLS include fibroblasts and stromal cells [52]. *It is worth noting that studies have shown that when growing in the lungs*,* pulmonary metastases from BC and renal cancer have a lower TLS density compared to those from lung cancer*,* prostate cancer*,* as well as CRC. Simultaneously*,* there is significant variability in TLS density from patient to patient. In the study*,* there were significant differences in the detection rates of TLS across six different types of cancer. Specifically*,* the detection rate of TLS in Esophageal Cancer was 40.0%*,* while in Gastric Cancer*,* the detection rate of TLS was as high as 75.6%* [83]. *Furthermore*,* in metastatic melanoma*,* different types of lymphoid aggregates have been found*,* with or without clearly defined B-cell or T-cell areas*,* and with or without the presence of HEV*,* FDC*,* and/or DC*,* indicating that TLS within tumors are heterogeneous* [42]. *This variability may affect the reliability and consistency of TLS as a prognostic marker*,* and thus may impact its application in clinical practice.* However, there is still a lack of consensus on how to measure TLS density and define what constitutes “high” or “low” density, which makes using TLS density as a standardized clinical marker of prognosis challenging. Further studies and data analysis are needed to translate TLS into a reliable clinical prognostic tool [70].

### Combined TLS and ICB treatment

The main goal of TLS induction is to transform ICB-resistant “cold” tumors into immune cell-infiltrated “hot” tumors, paving the way for successful ICB therapy. Currently, interferon gene stimulator (STING) agonists may be able to induce TLS [84]. Tumor cells typically exhibit high concentrations of cytoplasmic DNA due to defective expression/function of DNA repair proteins, which activate the cGAS-STING pathway. For example, treatment of melanoma with the STING agonist ADU S-100 increased the production of several TLS-inducible factors, such as CCL19, CCL21, Lt-α, Lt-β, and Light, and inhibited melanoma growth [84]. However, importantly, the TLS produced by this treatment were not classical TLS, as the homeostatic chemokine CXCL13, typically highly expressed in TLS, was not highly expressed after ADU S-100 treatment.

Cancer treatment can enhance the anti-tumor immune response of T cells and B cells by inducing TLS to increase lymphocyte infiltration [64, 85]. Such treatments include chemotherapy, radiotherapy, and immunotherapy based on ICBs. TLS induction may be crucial in rebuilding the body’s immunity after chemotherapy. Data from a cohort of children with APC-mutant hepatoblastoma revealed that after neoadjuvant chemotherapy with cisplatin, TLS were significantly present in all tumors, whereas no TLS were detected before chemotherapy. Similarly, clinical data from another cohort of patients with epithelioid mesothelioma who underwent neoadjuvant chemotherapy revealed a significant correlation between chemotherapy and TLS (*p* = 0.01). This suggests that some chemotherapeutic agents can induce TLS. Radiotherapy promotes local expression of *MHC-I* and co-stimulatory molecules and increases the number of CD8^+^ T cells around tumors. However, there is no clear evidence that radiotherapy induces TLS formation within tumors; hence, further studies are required.

In addition to serving as markers of therapeutic response, TLS can be used as immunotherapy targets. In cancers where TLS are associated with host protection, TLS can be induced locally by injecting relevant chemokines (CCL21, CCL19, and CXCL13), thus eliminating the need for therapeutic immunization against unknown antigens.TLS have emerged as significant components in the tumor microenvironment, playing a crucial role in the immune response against cancer. Recent studies have highlighted the potential of TLS as predictive biomarkers for immunotherapy response, particularly in melanoma. The presence of TLS in the tumor microenvironment has been associated with favorable responses to immunotherapy, characterized by improved survival rates and clinical outcomes. This suggests that TLS might be pivotal in tailoring more efficient and personalized treatments for individuals with melanoma [86].

Nowadays, researchers are particularly interested in the molecular mechanisms underlying TLS-ICB interactions. Many components secreted by cells in TLS, such as CXCL13, CCL19 [87], and other chemokines, may have their expression or signaling pathways affected by ICB, thereby altering the distribution of immune cells in TLS.It has been discovered that ICB can reverse the suppression of T cells by immune checkpoints and enhance T cell activity, allowing T cells to interact with other immune cells in the TLS and enhance the immune response, thereby recognizing and destroying tumor cells more effectively.

In many cancers, ICB induces antitumor immune responses and results in long-term response rates. However, not all patients respond; thus, identifying reliable response biomarkers is an urgent medical need. TLS, as the sites for generating circulating effector memory cells that control tumor recurrence, provide a unique opportunity to guide the next generation of clinical trials in the expanding field of immuno-oncology.

Several recent studies have provided evidence for the predictive value of TLS for ICB. The presence of TLS and active B cell infiltrates in the pre-treatment biopsies of melanoma, RCC, STS, and UC has been demonstrated to correlate with the response to *PD-1* inhibition or the combination of *PD-1* and CTLA-4 [64]. Similarly, pre-treatment biopsies from responders to melanoma ICB contain many TLS components. *In a checkpoint blockade-resistant tumor model in mice*,* combination therapies capable of inducing TLS-sensitized tumors to ICB and induced the production of effector and memory T cells*,* implying that TLS creates a TME that allows ICB to function* [88].

Meanwhile, analysis of tumor biopsies revealed that ICB treatment also promotes TLS formation. *In patients with high-risk melanoma and UC undergoing neoadjuvant ICB therapy*,* patients who responded show the enrichment of TLS-associated B cells* [63].Thus, ICB therapy increases the number and size of TLS and contributes to tumor control.

In a study of patients with melanoma, researchers collected melanoma biopsy samples from patients who underwent CTLA-4 blockade. The data revealed that tumors with higher levels of TLS exhibited significantly increased survival after CTLA-4 blockade. Additionally, in a previously published dataset of pre-treatment samples from 69 patients who received *anti-PD-1* monotherapy or a combination of anti-CTLA-4 and *anti-PD-1* therapy, TLS characteristics were significantly associated with overall survival [87]. The TLS signature exhibited a better predictive effect on the pre-treatment sample of 41 patients receiving *anti-PD-1* therapy [87]. ICB treatment induced a TLS response. This indicates that using PD-1 inhibitor therapy in combination with TLS or PD-1 inhibitor and CTLA-4 inhibitor in combination with TLS will produce synergistic effects, enhance the immune response, and effectively delay growth and spread to achieve better efficacy (Table 1).

**Table 1:** Detailed analysis of the efficacy of combination therapies for different Cancer types

Type	*Treatment measures*	*Potential/Predicted immune response changes*	Results	Ref.
B16 melanoma	Commenced with *anti-PD-1* antibody pre-treatment before combined administration	IFN-α and IFN-γ responses↑	Enhancing the CD8^+^ T cell/Treg ratio and amplifying antitumor immunity	[89]
metastatic melanoma	Combined Ipilimumab and a glycoprotein 100 (gp100) peptide vaccine	*N/A*	*A 19% reduction in the risk of progression was noted with ipilimumab plus gp100*,* as compared with gp100 alone*, but there’s aslo a 36% reduction in risk of progression with ipilimumab alone as compared with gp100 alone*. The overall survival all improved.	[90]
metastatic, squamous non-small-cell lung cancer	Pembrolizumab plus chemotherapy	The inhibition of T cells↓	Median OS prolonged by 4.6 months and median PFS by 1.6 months	[91]
melanoma	Nivolumab plus ipilimumab	The activation and proliferation of T cells↑	PFS, OS, and ORR↑	[92]
Pancreatic Neuroendocrine Tumor (PNET)	Combined LIGHT-VTP therapy and CTLA-4 and PD-1	Intratumoral activation of cytotoxic T cells↑	normalizes blood vessels, ensure survival benefit which can be further improved	[88]

Note PFS: progression-free survival, OS: overall survival, ORR: objective response rate

* From the data provided in the table, we can see that although combination therapy is beneficial for improving overall survival. But in this case, monotherapy with ipilimumab has a better effect than combination therapy.

Although ICB increases the number of TLS in several cancer types, it is unclear whether it can induce *de novo* TLS formation. Moreover, although the induction or enhancement of TLS function may improve tumor control, such interventions may concurrently enhance autoreactive T and B cell responses at other tissue sites. Combination therapy may result in an increased incidence and severity of immune-related adverse events, including arthritis, myositis, thyroiditis, vasculitis, and colitis, posing additional health risks [64]. Continuous exposure of TLS to tumor-associated autoantigens may promote the production of autoreactive antibodies. In ovarian cancer, TLS may contribute to paraneoplastic neurological syndromes (PNSs) affecting multiple neural tissues and structures [66]. Although PNS-associated cancers are very rare (< 1%) [66], the extensive use of ICBs may increase the risk of these syndromes. Given the variability in individual response to combination therapy, clinical vigilance is crucial. Nevertheless, the combination of ICB and TLS is promising. With a deeper understanding of tumor immunology and molecular biology, it can be hypothesized that TLS induction in tumors may lead to lymphocyte recruitment and enhanced tumor control. Combining TLS with ICIs can enhance immune cell infiltration, activation, and ability to destroy tumor cells, allowing for tumor treatment and advancing the field of immuno-oncology therapy.

### TLS improvements to irAEs

IrAEs can occur in any organ system due to the unintended ICI-mediated activation of the immune system. IrAEs occur as a result of increased specific toxicity of ICI therapy, with irAEs in anti-CTLA-4 drug therapy being dose-dependent, whereas irAEs in *anti-PD-1* drug therapy appear to be dose-independent. The skin, gastrointestinal, hepatic, endocrine, and pulmonary systems have a high incidence of irAEs, while cardiovascular irAEs are rare but have the highest risk of mortality [93].

T cells play a crucial role in the pathogenesis of most irAEs. The development of cutaneous irAEs may be associated with proinflammatory cytokines. For example, psoriasis is driven by activation of the Th17 pathway, with IL-23, IL-17 being highly potent in lesions. Atopic dermatitis is driven by Th2 cells, with elevated levels of IL-4, IL-5, IL-13, and IgE promoting inflammation. Furthermore, Ang-1 is elevated in psoriasis and Atopic dermatitis(AD), disrupting the epithelial barrier and increasing inflammation. irAEs may also be associated with B cells and antibodies; therefore, drugs targeting CD20 and B cells may be used to treat autoantibody-driven irAEs such as herpes-like varicose vein disease [94]. In mouse experiments, the pathogenesis of ICI-associated diabetes has been hypothesized to result from a lack of *PD-1*, resulting in an increase in autoreactive CD4^+^ and CD8^+^ T cells, leading to pancreatic islet cell destruction. This diabetes can be reversed by *PD-1* overexpression or pharmacologic restoration. Human studies have demonstrated that normal cells can upregulate *PD-1* expression to inhibit T cell function, and blocking this process with ICI treatment may lead to diabetes [95]. In studies of the pathogenesis of destructive thyroiditis, *anti-PD-1* inhibitors have been reported to increase the number of CD4^+^ T cells in the thyroid gland. *Cytotoxic CD4*^*+*^*T cells directly damage thyrocytes via recognition of thyroglobulin and MHC class II expressed on thyrocytes* [96]. The main neurologic manifestations of irAEs include encephalitis, aseptic meningitis, uillain-Barré syndrome (GBS), myasthenia gravis, and myositis. ICI-induced encephalitis is usually not associated with antisynaptic receptors or autoreceptors for neuronal cell surface proteins; however, cerebrospinal fluid levels of adenosine deaminase are elevated. Patients with irAE-type GBS typically exhibit an increase in cerebrospinal fluid lymphocytes and nerve root hypertrophy on spinal magnetic resonance imaging. Myasthenia gravis is caused by dysfunction of the neuromuscular junctions of skeletal muscle, occurs early in ICI therapy, and rapidly deteriorates. Detection of myasthenia gravis requires screening for anti-ACHR antibodies. Anti-striatal may indicate an overlap between myasthenia gravis and myositis. ICI treatment can activate the cytotoxicity of autoreactive CD8^+^ T cells, leading to the production of anti-ACHR and anti-striatal [97].

In a study of gastric cancer, nivolumab induced more irAEs in patients with high TLS; however, these patients also experienced better treatment outcomes and prognosis [98]. Research on Tfh cells has demonstrated that Tfh cells can secrete CXCL13, and the associated genetic profile is considered a predictor of TLS activity. In patients with melanoma treated with anti-PD-1 therapy, irAEs may result from cTfh cell dysregulation, increased expression of the cTfh cell proliferation-related pathway, and increased CXCL13 secretion. Thus, we hypothesized that TLS could predict the development of irAEs; however, the relationship needs to be further investigated [75]. In a study of renal irAEs, the diagnosis of acute interstitial nephritis (AIN) associated with TLS was investigated. Currently, renal biopsy is the common method for diagnosing AIN; however, it carries the risk of hemorrhage and presents diagnostic challenges. This work proposes using TLS signaling in irAEs to distinguish ICI-related AIN from other chemokines commonly associated with acute kidney injury. This study discovered that the 12-chemokine TLS gene signature signals (CCL2, CCL3, CCL4, CCL5, CCL8, CCL18, CCL19, CCL21, CXCL9, CXCL10, CXCL11, and CXCL13) were significantly upregulated in the AIN group, with CXCL9 and CXCL10 being detectable in urine at levels comparable to those in renal tissue. CXCL9 and CXCL10 were closely correlated with AIN identification (Table 2) [99].

Current research indicates that the molecular mechanism underlying ICB-induced TLS generation remains unclear. An in-depth study of the interaction between ICB and TLS will help to identify more precise therapeutic targets. Analyzing the composition, structure, and biomarkers of TLS can help identify patient groups that are more susceptible to ICB and the synergistic treatment of TLS and ICB and optimize therapeutic decisions. Additionally, the new biomarkers, combined with the structural and functional characteristics of TLS, allow for more accurate prediction of the effects of treatment regimens and the occurrence of irAEs, which can help mitigate risks and improve treatment safety. Monitoring the dynamic changes in TLS after combination therapy may improve the success rate of the treatment regimen and provide novel insights for future research and clinical treatments.

**Table 2:** Multiple roles between TLS and ICB

Type	Cancer category	Intervention	Changes in TLS	Results	Ref.
Prediction	NSCLC	Inhibitors of *PD-1/PD-L1* signaling axis	↑	PD-L1 blockade↑	[78]
	UC	*Anti-PD1/*CTLA-4 checkpoint inhibitors	↓	Unresponsive immunotherapy	[82]
	RCC	*PD1* blockade monotherapy versus combined CTLA-4 and *PD1* blockade versus combined PD1 blockade and bevacizumab	↑	B-cell-related exosomes (CD20^+^)↑ expression of B-cell-related genes↑	[46]
	SIC E	*Anti-PD1* monoclonal antibody pembrolizumab	↑	ORR↑	[80]
	soft-tissue sarcoma	*PD-1* antagonist	↑	A high expression of B cell lineage genes	[100]
Induction	UC	*Anti-PD1/*CTLA-4 checkpoint inhibitors	↑	Early TLS enrich	[82]
	STS	pembrolizumab combined with low-dose cyclophosphamide	↑	Dense PC infiltrates	[101]
	Melanoma	*CTLA4* blockade	↑	TLS signature↑	[87]
	WT mice TNFR1/2 mice OC NCT02626130 Head and neck cancer Resectable malignant pleural mesothelioma	*Anti-PD-L1* monotherapy or the combination of anti-CTLA-4 and *anti-PD1.* *Anti-PD-L1* monotherapy or the combination of anti-CTLA-4 and *anti-PD1* guadecitabine and pembrolizumab Anti-CTLA-4 with cryoablation Ad5/3 Ad5 and ICI Durvalumab plus tremelimumab	↑ → ↑ ↑ ↑ ↑	I.P. tumor size↓ S.C. B16-OVA tumor size→ tumor size→ Classical monocytes and central memory CD4^+^ T cells↑ Immune cell infiltration↑ Tumor growth control↑ Remodeled the immune contexture of MPM tumors	[102] [102] [103] [104] [105] [106]
	Melanoma	Anti-CTLA-4 followed by *anti-PD-1* therapy	↑	Efficacy of anti-PD-1 treatment↑	[107]
Correlation	AIN	NanoString-based gene expression and multiplex 12-chemokine profiling	↑	Urine chemokine markers may be used as a surrogate for ICI-AIN diagnoses	[99]
	irAE incidence	Measurement of cytokines, serologic clinical markers, and Ig	↑	A systemic increase in CXCL13 and IL-21 expression in CD4 T cells correlated with irAE incidence	[108]
	Melanoma	Undergoing CPI therapy	*N/A*	CPI-induced B cell changes positively correlated with frequency and severity of irAEs	[109]
	SLE	RNA-Seq confirmed by RT-qPCR	↑	CXCL13 and CCL19 are likely effectors in the recruitment and organization of leukocytes in the formation of a TLS in NPSLE	[110]
Combination	HNSCC	Combinatorial treatment with *anti-PD-1* or *anti-PD-L1* blocking mAb and Adenovirus Encoding TNFα and IL − 2	↑	Enhance both the adaptive cell immune response through stimulation of cytotoxic T cells and the humoral response through generation of antitumor antibodies	[105]
	Pten-null prostate cancer	Intermittent dosing of a PI3Kα/β/δ inhibitor, BAY1082439	↑	Alleviate PTEN-null cancer cell-intrinsic immunosuppressive activity	[111]
	Anaplastic thyroid cancer (ATC)	Famitinib plus *anti-PD-1(aPD-1)* immunotherapy	↑	The formation of HEVs↑ induces mature TLS development and restricts tumor growth in patients with ATC	[112]
	NSCLC	Radiotherapy combined with anti-CTLA-4 therapy	↑	The median overall survival ↑	[113]

Note: Prediction: TLS plays a predictive role in the treatment of ICB; Induction: ICB can induce the formation of TLS. Correlation: TLS correlates with immune adverse events; Combination: TLS can be combined with ICB for cancer treatment. Correlation: TLS correlates with immune adverse events; Combination: TLS can be combined with ICB for cancer treatment. →: *remain unchanged*

## Conclusion

TLS, which are key sites for immune cell activation and proliferation, provide a suitable microenvironment for tumor immune response, promote immune cell activation, proliferation, and infiltration, and support antitumor immune responses through mechanisms such as antigen presentation, cytokine secretion, and immune cell recruitment. ICB therapy reduces tumor-induced immunosuppression by blocking immune checkpoint molecules, such as *PD-1/PD-L1* and CTLA-4, allowing immune cells to exert their antitumor activity.

Studies have demonstrated that combining ICB and TLS provides significant advantages. Immune cell activation and proliferation in TLS provide more active immune cell targets for ICB treatment. Meanwhile, ICB treatment enhances the function of immune cells in TLS and promotes a more effective immune response.

However, there are some limitations in this study. For example, the reliability of TLS as prognostic markers remains uncertain. The long-term efficacy and potential adverse effects of ICB therapy require further observation. Besides, more clinical studies are needed to determine the optimal regimen and applicable populations for combination therapy.

Nevertheless, ICB and TLS combination therapy has introduced new avenues in tumor immunotherapy. Future studies should focus on revealing the molecular details of their synergistic effects, developing more precise therapeutic strategies, optimizing treatment regimens, and combining emerging biotechnology and multi-omics research methods to achieve more personalized and effective tumor therapy, giving patients with tumors more hope for survival and a better quality of life.

## Data Availability

All data generated or analyzed in this work are included in this article and/or its figures. Further enquiries can be directed to the corresponding author.

## Abbreviations

ICB: Immune checkpoint blockade
TLS: Tertiary lymphoid structures
NSCLC: Non-small cell lung cancer
ICIs: Immune checkpoint inhibitors
CTLA-4: Cytotoxic T lymphocyte-associated protein 4
irAEs: Immune-related adverse events
TME: Tumor microenvironment
MHC: Major histocompatibility complex
APCs: Antigen-presenting cells
PD 1H: PD-1 homolog
TCR: T cell receptor
LN: Lymph nodes
FDCs: Follicular dendritic cells
GCs: Germinal centers
HEVs: High endothelial venules
LAMP^+^: Lysosome-associated membrane protein
RA: Rheumatoid arthritis
ILCs: Innate lymphoid cells
LTBR: Lymphotoxin beta receptor
SLOs: Secondary lymphoid organs
BCRs: B cell receptors
Tfh: T follicular helper cells
TMB: Tumor mutational burden
CRC: Colorectal cancer
PFS: Progression-free survival
OS: Overall survival
HCC: Hepatocellular carcinoma
BC: Breast cancer
RCC: Renal cell carcinoma
H&E: Hematoxylin and Eosin
PCs: Plasma cells
UC: Uroepithelial carcinoma
STS: Soft tissue sarcomas
SIC E: Sarcoma Immune Classes E
PVTT: Portal vein tumor thrombosis
TIME: Tumor immune microenvironment
STING: Interferon gene stimulator
PNET: Pancreatic Neuroendocrine Tumor
LLC: Lewis lung carcinoma
PNSs: Paraneoplastic neurological syndromes
AD: Atopic dermatitis
GBS: Uillain-Barré syndrome
AIN: Acute interstitial nephritis
